# CRISPR/Cas9 Ribonucleoprotein Delivery Enhanced by Lipo-Xenopeptide Carriers and Homology-Directed Repair Modulators: Insights from Reporter Cell Lines

**DOI:** 10.3390/ijms26094361

**Published:** 2025-05-03

**Authors:** Xianjin Luo, Eric Weidinger, Tobias Burghardt, Miriam Höhn, Ernst Wagner

**Affiliations:** 1 Pharmaceutical Biotechnology, Department of Pharmacy, Ludwig-Maximilians-Universität Munich, Butenandtstrasse 5-13, 81377 Munich, Germany; xianjin.luo@cup.uni-muenchen.de (X.L.); eric.weidinger@cup.uni-muenchen.de (E.W.); tobias.burghardt@cup.uni-muenchen.de (T.B.); miriam.hoehn@cup.uni-muenchen.de (M.H.); 2 Center for Nanoscience (CeNS), LMU Munich, 80799 Munich, Germany; 3 CNATM—Cluster for Nucleic Acid Therapeutics Munich, 81377 Munich, Germany

**Keywords:** CRISPR Cas9, Cas9 ribonucleoprotein, Cas9 mRNA/sgRNA, homology-directed repair, enhancer, cell cycle

## Abstract

CRISPR-Cas9 genome editing is a versatile platform for studying and treating various diseases. Homology-directed repair (HDR) with DNA donor templates serves as the primary pathway for gene correction in therapeutic applications, but its efficiency remains a significant challenge. This study investigates strategies to enhance gene correction efficiency using a T-shaped lipo-xenopeptide (XP)-based Cas9 RNP/ssDNA delivery system combined with various HDR enhancers. Nu7441, a known DNA-PKcs inhibitor, was found to be most effective in enhancing HDR-mediated gene correction. An over 10-fold increase in HDR efficiency was achieved by Nu7441 in HeLa-eGFPd2 cells, with a peak HDR efficiency of 53% at a 5 nM RNP concentration and up to 61% efficiency confirmed by Sanger sequencing. Surprisingly, the total gene editing efficiency including non-homologous end joining (NHEJ) was also improved. For example, Nu7441 boosted exon skipping via NHEJ-mediated splice site destruction by 30-fold in a DMD reporter cell model. Nu7441 modulated the cell cycle by reducing cells in the G1 phase and extending the S and G2/M phases without compromising cellular uptake or endosomal escape. The enhancement in genome editing by Nu7441 was widely applicable across several cell lines, several Cas9 RNP/ssDNA carriers (LAF-XPs), and also Cas9 mRNA/sgRNA/ssDNA polyplexes. These findings highlight a novel and counterintuitive role for Nu7441 as an enhancer of both HDR and total gene editing efficiency, presenting a promising strategy for Cas9 RNP-based gene therapy.

## 1. Introduction

Genome engineering using nucleases offers a powerful platform for generating both gene knock-outs and knock-ins through the repair of DNA double-strand breaks (DSBs) [1,2]. These genetic modifications rely on two primary pathways: non-homologous end joining (NHEJ) and homology-directed repair (HDR) [3]. For gene knock-outs, the error-prone NHEJ pathway functions without a template, introducing random insertions or deletions [4]. In contrast, HDR is essential for precise gene correction in therapeutic applications, enabling accurate repair and template-guided insertions [5,6,7,8,9,10,11]. Additionally, emerging Cas9-based approaches, such as base editing [12,13] and prime editing [13,14], facilitate targeted gene modifications and small insertions (<50 bases) without requiring DSBs. However, unlike base and prime editing, HDR-mediated editing is not constrained by sequence length and supports insertions spanning several kilobases [15], making it the most versatile method for achieving complex targeted substitutions and insertions. Despite its advantages, CRISPR/Cas9 HDR efficiency remains relatively low in standard editing conditions and far lower as compared to NHEJ-mediated editing, posing a significant challenge to its widespread application. As a result, the development of effective delivery platforms is critical to advancing HDR-mediated gene therapy.

Traditional CRISPR/Cas9 delivery methods, such as microinjection and electroporation, are effective for in vitro applications but often cause cell membrane damage due to the need for individual cell manipulation. These methods are also less suitable for tissue-level or in vivo applications [16,17]. In contrast, viral and non-viral vectors present promising alternatives for delivering Cas9 in both in vitro and in vivo contexts. Viral vectors, such as lentiviral, adenoviral, and adeno-associated viral vectors, have been developed as gene editing tools by researchers like Merienne [18], Xu [19], and Ibraheim [20]. However, these vectors are constrained by cargo size limitations and the risk of immune activation [21]. Non-viral delivery systems, on the other hand, are emerging as versatile platforms with broad applications and are poised to dominate future delivery strategies [22,23,24,25,26,27,28,29,30,31,32,33]. For instance, Zhang et al. developed lipid-based Cas9 mRNA nanoparticles that effectively knocked out PD-L1 in tumors, significantly reducing tumor growth and metastasis in a mouse model [34]. Similarly, Tan et al. engineered a dual-functionalized polymer incorporating boronate and lipoic acid moieties, which served as a Cas9 RNP carrier targeting NLRP3, successfully disrupting inflammasomes and mitigating inflammation in a psoriasis mouse model [35]. Building on the chemical evolution of sequence-defined xenopeptides (XPs) generated by solid phase-supported synthesis [36,37,38], our previous work introduced synthetic T-shaped lipo-XPs as CRISPR/Cas9 RNP carriers with high gene editing and correction potential. Notably, the lipo-XP *ID 1738* (dGtp)- and *ID 1636* (Gtt)-based Cas9 nanocarriers demonstrated high delivery efficiency, with the *1636*-based RNP complex achieving an HDR efficiency of 40% [39].

Furthermore, studies have demonstrated that various HDR enhancers based on small molecules have been utilized to improve CRISPR/Cas9 HDR-mediated gene editing efficiency. These enhancers include NHEJ pathway inhibitors, HDR pathway stimulators, and cell cycle synchronization agents [40]. The NHEJ pathway is triggered by DSBs, which recruit the Ku70/80 heterodimer and the catalytic subunit of DNA-dependent protein kinase (DNA-PKcs) [41,42]. Together, these components tether the DNA ends, initiating a cascade of reactions including resection, extension, and ligation. Inhibiting key factors of the NHEJ pathway, such as Ku70/80 [43], DNA-PKcs [44], and ligase IV [45], has proven to be an effective strategy for enhancing HDR. For instance, the DNA-PKcs inhibitors Nu7441 and KU-0060648 have been shown to significantly increase HDR repair events while reducing NHEJ frequency in Cas9-mediated gene editing [46]. Similarly, SCR7, a ligase IV inhibitor, has achieved up to a 19-fold improvement in HDR efficiency when combined with Cas9 in mammalian cell lines and mouse models across four target genes [45]. On the HDR pathway side, RAD51, a key factor in DNA strand exchange, plays a central role by forming presynaptic filaments with the accumulation of other proteins on single-stranded DNA (ssDNA). Rs-1, identified from a screen of 10,000 compounds, enhances RAD51 binding to DNA, thereby stimulating HDR [47]. In this study, we primarily investigated strategies to improve HDR efficiency using both NHEJ pathway inhibitors and HDR pathway stimulators in combination with T-shaped peptide-based Cas9 RNP complexes. The evaluated enhancers include Pevonedistat, Rs-1, M3814, KU-0060648, and Nu7441.

Overall, we developed an improved gene correction and editing platform for treating genetic disorders. This platform integrates one of the top T-shaped lipo-XP candidates, in combination with the DNA-PKcs inhibitor Nu7441. Remarkably, this system enhanced both knock-in-mediated gene correction and knock-out-based total gene editing efficiency. Mechanistically, Nu7441 modulated the cell cycle by inhibiting the G1 phase and extending the S and G2/M phases without compromising cellular uptake or endosomal escape. In GFP-to-BFP conversion models, the platform achieved a more than 10-fold increase in HDR efficiency with Nu7441 treatment. The HDR-mediated gene editing efficiency reached 53% in flow cytometry analysis when using the *1636* (Gtt)-based Cas9 nanocarriers. Notably, gene sequencing confirmed that the HDR efficiency peaked at 61%. Beyond HDR enhancement, Nu7441 also amplified exon skipping by 30-fold in a DMD exon 23 reporter cell model and exhibited broad compatibility across various cell lines and LAF-XP oligomers for gene correction and editing. These findings highlight the platform’s significant potential as an efficient and versatile tool for future gene therapy applications.

## 2. Results

### 2.1. Characterization of T-Shaped Cas9 RNP/ssDNA Complexes and Selection of Enhancers

**Figure 1:**
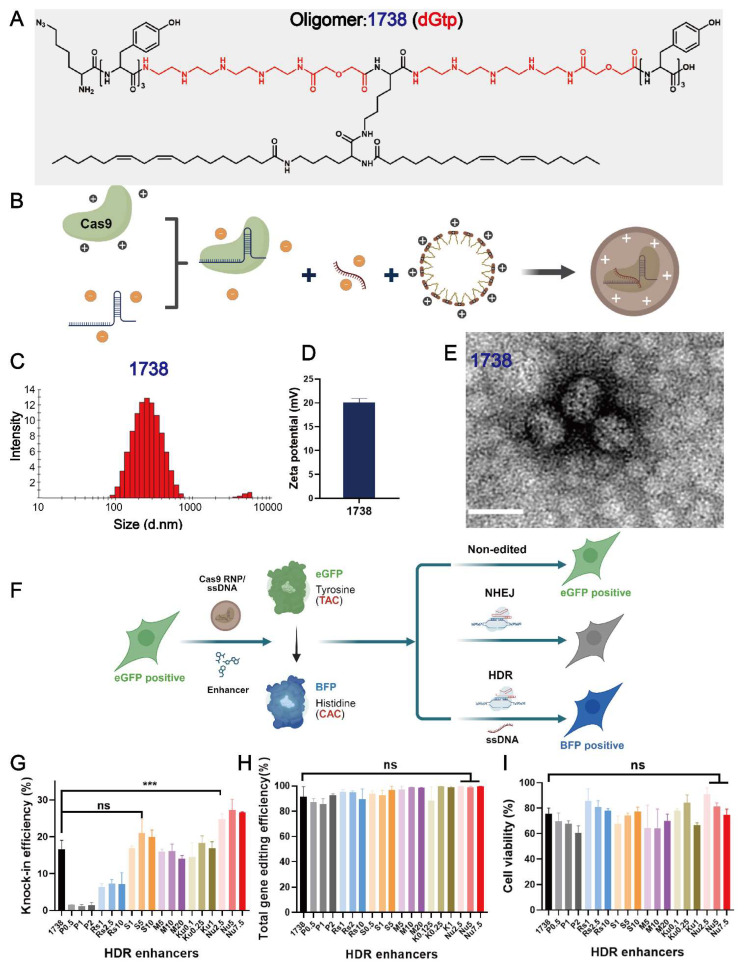
Characterization of Cas9 RNP complexes and selection of gene correction enhancers. (**A**) The chemical structure of T-shaped lipo-XP *1738* (dGtp). (**B**) Schematic illustration depicting T-shaped lipo-XP-based Cas9 RNP complexes prepared by mixing Cas9 protein with sgRNA, ssDNA, and lipo-XP *1738*. Dynamic light scattering (DLS) intensity size distribution (**C**) and zeta potential (**D**) of *1738*-based Cas9 RNP/ssDNA complexes at an RNP/ssDNA ratio of 1:1 and N/P = 12. (**E**) Transmission electron microscopy (TEM) image of *1738*-based Cas9 RNP/ssDNA complexes at an RNP/ssDNA ratio of 1:1 and N/P = 12 (scale bar, 80 nm). (**F**) Schematic illustration of eGFP-to-BFP conversion in eGFPd2 cells. GFP expression can be eliminated via NHEJ, or the 66th amino acid, tyrosine (TAC), can be changed to histidine (CAC) through HDR to produce BFP expression. (**G**) HDR efficiency, (**H**) total gene editing efficiency (both HDR and NHEJ events), and (**I**) cell viability of HeLa-eGFPd2 cells treated with *1738*-based Cas9 RNP/ssDNA complexes (18.75 nM, RNP) with various gene correction enhancers, including Pevonedistat (0.5, 1, 2 nM), Rs-1 (1, 2.5, 10 nM), SCR7 (1, 5, 10 nM), M3814 (5, 10, 20 nM), KU-0060648 (0.1, 0.25, 1 nM), and Nu7441 (2.5, 5, 7.5 nM). “*1738”* refers to treatment with *1738* Cas9 RNP/ssDNA complexes without enhancer. Enhancer abbreviations represent the enhancer name followed by its concentration in nM. *** *p* < 0.001 vs. *1738*; ns denotes no significant difference. Data are shown as means ± SD *(n = 3*), with statistical significance determined by unpaired Student’s *t*-test.

In this study, to identify various HDR enhancers, a recently designed gene editing T-shaped lipo-XP, ID *1738* (dGtp), was selected as the main delivery carrier (Figure 1A). This oligomer demonstrated excellent gene editing efficiency with minimal cellular toxicity. Lipo-XP *1738* has the peptidic sequence (N- to C-terminus) K(N_3_)-Y_3_-dGtp-K(K(LinA)_2_)-dGtp-Y_3_. It is composed of C- and N-terminal tyrosine tripeptides and central branching lysines as natural amino acids, modified with two linoleic acids in a T-shaped configuration, and two units of the artificial amino acid diglycoloyl tetraethylene pentamine (dGtp). Lipo-XP *1738* was synthesized using standard Fmoc-based solid-phase peptide synthesis, purified, and characterized as previously described [39]. The T-shaped Cas9 RNP/ssDNA complexes (Figure 1B) were then prepared by mixing *1738* and Cas9 RNP/ssDNA at a nitrogen-to-phosphate (N/P) ratio of 12, with an RNP/ssDNA ratio of 1:1, followed by 40-min incubation. The resulting nanoparticles had a hydrodynamic size of 150 nm (Figure 1C) and a zeta potential of +20.1 mV (Figure 1D). To visualize the Cas9 RNP/ssDNA complexes, the sample was stained with 5 μL of 1.0% uranyl formate solution, dried for 15 min, and analyzed using a JEOL JEM-1100 electron microscope. The results revealed a spherical shape with a diameter of 50–60 nm (Figure 1E).

The *1738*-based Cas9 RNP/ssDNA complexes were evaluated in a GFP-to-BFP conversion model (Figure 1F) using previously described HeLa-eGFPd2 cells [39]. The applied sgRNA, ssDNA, and reporter gene sequences are listed in the Supplementary Materials Table S1. To identify potential compounds that could further enhance the genome correction efficiency, we screened a range of small-molecule modulators targeting either the HDR or NHEJ pathways in HeLa-eGFPd2 cells. The cells were treated with *1738*-based Cas9 RNP/ssDNA complexes (18.75 nM) in the absence (“*1738*”) or presence of selected enhancers for 24 h.

The tested compounds included HDR pathway stimulators—Pevonedistat (CtIP stimulator; 0.5, 1, 2 nM) [48] and RS-1 (RAD51 stimulator; 1, 2.5, 10 nM) [49,50]—as well as NHEJ pathway inhibitors—SCR7 (ligase IV inhibitor; 1, 5, 10 nM) [51,52,53] and three DNA-PKcs inhibitors: M3814 (5, 10, 20 nM) [11,54], KU-0060648 (0.1, 0.25, 1 nM) [46,55], and Nu7441 (2.5, 5, 7.5 nM) [46,56,57]. Following treatment, the cells were washed with PBS and incubated for an additional five days in fresh medium. Flow cytometry analysis identified eGFP-positive (non-edited), eGFP-negative (NHEJ), and BFP-positive (HDR) populations (Figure 1F). Among the enhancers tested, only Nu7441 significantly improved the HDR efficiency, increasing it from 16.5% in the *1738* nanocarrier group to around 25% (Figure 1G), without a substantial difference in the total gene editing efficiency (both HDR and NHEJ events) (Figure 1H). Additionally, all enhancers exhibited minimal cytotoxicity (Figure 1I). Based on these findings, Nu7441 was selected for further development in subsequent experiments.

### 2.2. HDR and Gene Editing Efficiency with Various T-Shaped XPs or Enhancer Combinations

**Figure 2:**
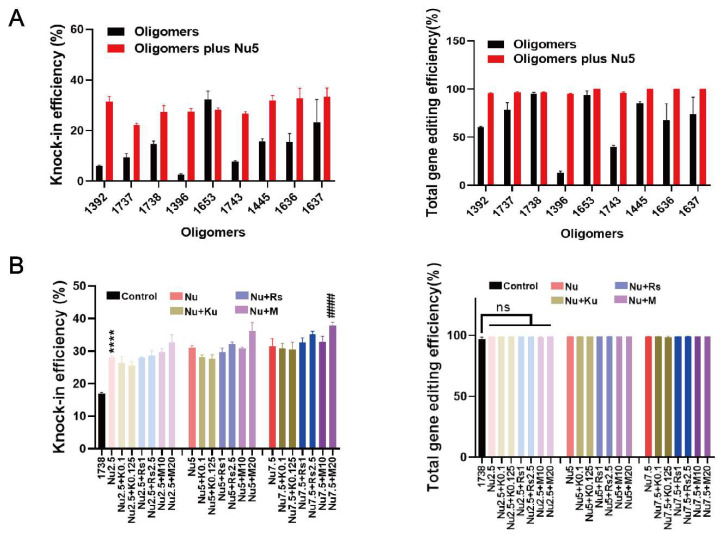
HDR and gene editing efficiency of Cas9 RNP/ssDNA complexes formed with nine different lipo-XP carriers and tested without or with enhancers in HeLa-GFPd2 cells. (**A**) HDR efficiency and total gene editing efficiency of T-shaped oligomer-derived Cas9 RNP/ssDNA complexes (18.75 nM RNP), transfected with or without 5 nM Nu7441. (**B**) HDR efficiency and total gene editing efficiency of *1738* Cas9 RNP/ssDNA complexes (18.75 nM RNP), in combination with enhancer mixtures, added for 24 h transfection. Enhancer mixtures include Nu7441 (2.5, 5, 7.5 nM), Rs1 (1, 2.5 nM), KU-0060648 (0.1, 0.25 nM), or M3814 (10, 20 nM). “*1738*” refers to treatment with *1738* Cas9 RNP/ssDNA complexes without enhancer. Enhancer abbreviations represent the enhancer name followed by its concentration in nM. **** *p* < 0.0001 vs. *1738*; #### *p* < 0.0001 vs. group Nu2.5; ns denotes no significant difference. Data are shown as means ± SD (*n* = 3), with statistical significance determined by unpaired Student’s *t*-test.

To further investigate the effects of Nu7441, the gene editing efficacies (knock-in and knock-out) of nine recently developed T-shaped lipo-XPs minus/plus Nu7441 were compared in HeLa eGFPd2 cells (Figure 2A). These carriers (Table S2) are derived from three lipo-XPs (*1392*, *1396*, and *1445*) with the artificial amino acid succinoyl tetraethylene pentamine (Stp) in their backbone. Within a subsequent chemical evolution process, carriers were further optimized by the replacement of Stp by other artificial amino acids including chGtp, dGtp, GEIPA, TFE, or Gtt (see Table S2), as recently published [39]. After 24 h of treatment, the cells were washed with PBS and incubated with fresh medium for an additional five days. The results demonstrated that 5 nM Nu7441 improved the BFP conversion efficiency for all T-shaped XPs, except for the already highly potent *1653* (Figure 2A). Notably, the HDR efficiency was increased by more than tenfold by the addition of Nu7441 to RNP/ssDNA complexes formed with *1396*. Interestingly, Nu7441 also increased the total gene editing efficiency of T-shaped lipo-XPs. Specifically, in the *1396* group, the total gene editing efficiency increased by approximately 7-fold, from 13.1% to 95.1%. These findings suggest that the DNA-PKcs inhibitor Nu7441 surprisingly also enhances NHEJ-mediated gene editing efficiency.

In addition, studies have shown that combining various enhancers represents an effective strategy for improving gene correction [9,48,58]. In our study, Rs1, KU-0060648, and M3814 at various concentrations were co-incubated with Nu7441 and *1738*-based Cas9 RNP/ssDNA complex for 24 h. After an additional five days of incubation, the cells were analyzed. The results indicate that the enhancer combination improved the HDR efficiency beyond the *1738*/Nu7441 group, resulting in nearly 40% BFP-positive cells with the combination of 7.5 nM Nu7441 and 20 nM M3814. The total gene editing efficiency reached almost 100% (Figure 2B). However, cell viability, assessed via an MTT assay after one day of incubation, revealed increased cytotoxicity (Figure S1). Consequently, Nu7441 alone, without additional enhancers, was identified as the preferable option for further development in subsequent studies.

### 2.3. Mechanistic Investigation of Nu7441 for Gene Editing Improvement

**Figure 3:**
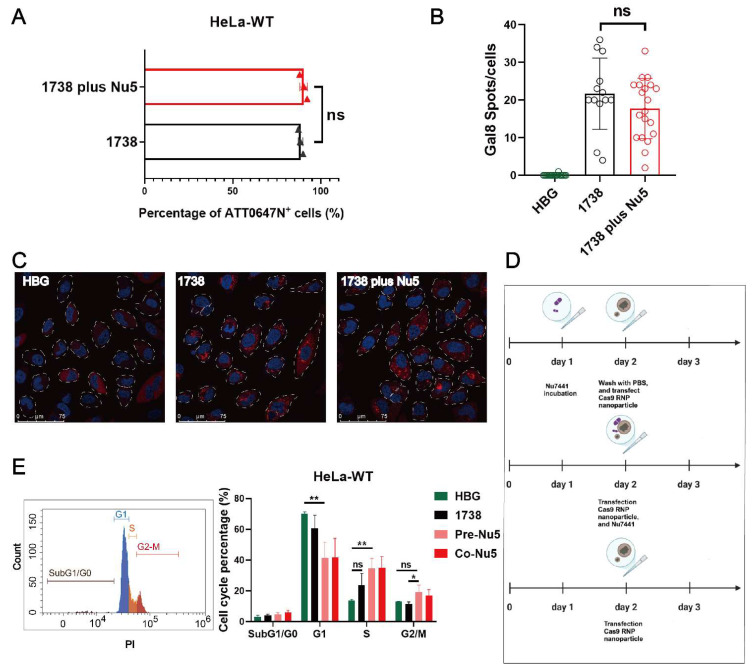
Mechanistic investigation of the Nu7441 effect. (**A**) Cellular uptake of *1738* Cas9 RNP/ssDNA complexes (37.5 nM) containing 20% ATTO647N-labeled Cas9 protein, with or without 5 nM Nu7441, in HeLa cells, analyzed by flow cytometry after 2 h of incubation. (**B**) Quantification and (**C**) confocal laser scanning microscopy (CLSM) imaging of gal8 puncta in HeLa gal8-mRuby3 cells treated with HBG or Cas9 RNP/ssDNA nanoparticles (37.5 nM), with or without 5 nM Nu7441 for 4 h. Nuclei were stained with DAPI (blue), while red punctate gal8-mRuby3 fluorescence indicates endosomal membrane disruption. Gal8 puncta were quantified using ImageJ analysis. (**D**) Experimental design for cell cycle analysis. (**E**) PI-based cell cycle analysis of HeLa cells treated with various *1738*-based nanocarriers (18.75 nM Cas9 RNP), analyzed on day 3 after treatment. Data are shown as means ± SD (*n* = 3), with statistical significance determined by unpaired Student’s *t*-test. * *p* < 0.05; ** *p* < 0.01; ns denotes no significant difference.

To further investigate the mechanism of Nu7441 in the gene editing process, cellular uptake experiments were conducted in standard HeLa cells. The cells were treated with *1738*-based Cas9 RNP/ssDNA complexes containing 37.5 nM RNP (20% ATTO647N- labeled Cas9 protein) for 2 h, with or without 5 nM Nu7441. The results demonstrated no significant difference in cellular uptake between the *1738*/Nu7441 and *1738* alone groups (Figure 3A), suggesting that Nu7441 does not influence cellular uptake. Similarly, endosome destabilization was analyzed in HeLa gal8-mRuby3 cells [38,59] as previously described [60]. After a 4-h treatment with HBG, *1738*-based Cas9 RNP/ssDNA complexes, or *1738* plus 5 nM Nu7441, the cells were stained with DAPI, and endosomal rupture was detected by CLSM. Upon endosomal disruption, cytosolic galectin-8 translocates to the damaged endosomes, where it binds to membrane-associated galactan residues [61,62]. The images revealed comparable gal8-mRuby3 fusion protein signals (red spots) in both groups (Figure 3B,C), indicating that Nu7441 does not alter endosomal escape activity.

Next, a PI-based cell cycle analysis was performed to investigate whether the gene editing enhancement by Nu7441 is associated with alterations in the cell cycle (Figure 3D). The cells were treated with *1738*-based Cas9 RNP/ssDNA complexes (18.75 nM) and 5 nM Nu7441 for various incubation methods (Figure 3D). Flow cytometry analysis revealed that Nu7441 significantly increased the proportion of cells in the S and G2/M phases while decreasing the G1 phase population (Figure 3E). The cell cycle was further analyzed on day 6 using standard transfection protocols in HeLa-eGFPd2 cells. The results were consistent with the above findings, showing a similar cell cycle distribution (Figure S2). Previous studies have shown that HDR is predominantly active in the S and G2 phases, coinciding with the availability of homologous sequences that can serve as repair templates during DNA damage [63,64]. Additionally, the G2/M stage has previously been demonstrated to facilitate transfection due to the breakdown of the nuclear membrane during the M phase and easier access to the nuclear genomic compartment [65,66,67,68]. To investigate whether Nu7441 can facilitate such a nuclear entry process, an mCherry mRNA and GFP pDNA co-transfection experiment was carried out with or without Nu7441 (5 nM). The results (Figure S3) demonstrated that Nu7441 enhanced pDNA transfection (which requires nuclear entry) but reduced mRNA transfection (where nuclear entry is not desired).

### 2.4. Exon Skipping Efficiency of 1738 Cas9 RNP Complexes with 5 nM Nu7441 in DMD Exon 23 Reporter Cell Model

**Figure 4:**
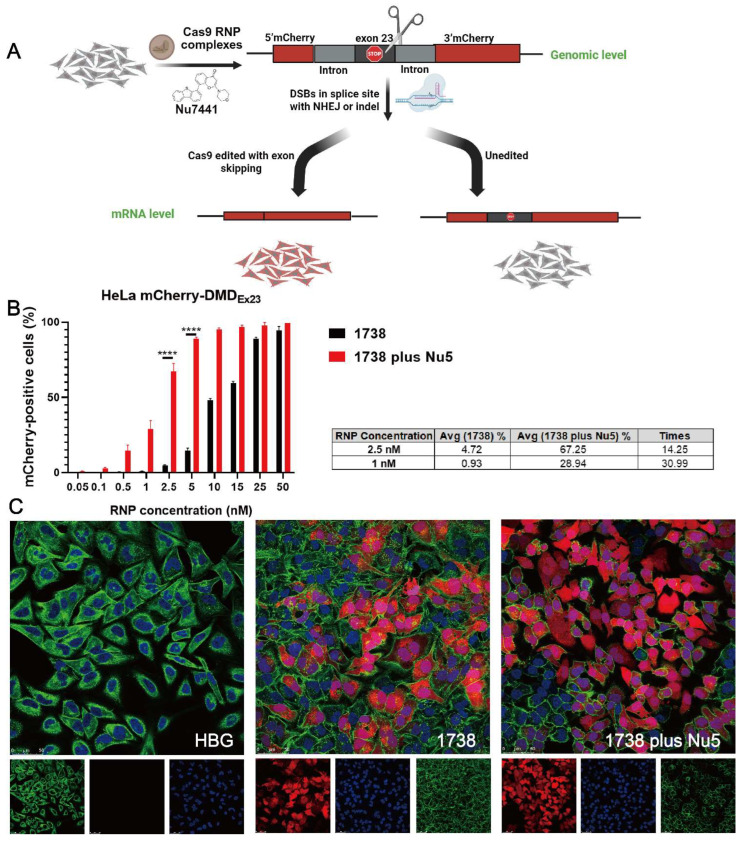
Exon skipping efficiency of Cas9 RNP complexes with NU7441 in a DMD reporter cell model. (**A**) Schematic representation of the exon 23 skipping mechanism using Cas9 RNP complexes in the absence or presence of 5 nM Nu7441. (**B**) Comparison of exon skipping efficiency between single *1738* Cas9 RNP complexes and the group supplemented with 5 nM Nu7441. Cas9 RNP concentrations ranged from 0.05 to 50 nM. The column chart illustrates significant improvements in exon skipping efficiency with Nu7441. (**C**) CLSM images of HeLa mCherry-DMD_Ex23_ cells treated with *1738* Cas9 RNP complexes at 10 nM RNP, either with or without 5 nM Nu7441. Cell nuclei stained with DAPI (blue), the cytoskeleton with Alexa Fluor^TM^ 488 phalloidin (green), and mCherry fluorescence (red). The scale bar represents 50 μm. All complexes were prepared at a N/P ratio of 24 and transfected for 24 h. Data are shown as means ± SD (*n* = 3). Statistical significance is indicated as **** *p* < 0.0001.

In our previous work, HeLa mCherry-DMD_Ex23_ cells [69] were validated as a DMD exon 23 reporter cell model, demonstrating high exon skipping efficiency using LAF-XPs Cas9 mRNA/sgRNA [70] or Cas9 protein/sgRNA complexes [60] even in serum-containing conditions and at a low sgRNA concentration. Exon skipping at the mRNA level was also confirmed in these studies by Germer et al. [70] and Lessl et al. [69]. The exon skipping mechanism is illustrated in Figure 4A. To assess whether Nu7441 enhances exon skipping, HeLa mCherry-DMD_Ex23_ cells were treated with *1738*-based Cas9 RNP nanocarriers at varying concentrations (0.05–50 nM), with or without 5 nM Nu7441, for 24 h. After treatment, the cells were washed with PBS and incubated in fresh medium for an additional two days. Flow cytometry analysis was subsequently performed to distinguish between mCherry-negative cells (non-edited, negative cells) and mCherry-positive cells (edited, red cells), evaluating the exon skipping efficiency under the experimental conditions.

The data revealed that the EC50 of exon skipping for the single *1738* group was 11.18 nM RNP, whereas the EC50 for the *1738* plus Nu7441 group significantly decreased to 1.65 nM, as shown in Figure S4. These results indicated that Nu7441 markedly enhanced exon skipping in the DMD model. Similarly, the exon skipping efficacies in the *1738* group reached 0.93% at 1 nM RNP and 4.72% at 2.5 nM RNP, whereas additional treatment with Nu7441 resulted in 28.94% and 67.25%, reflecting 29.99-fold and 13.25-fold improvements, respectively (Figure 4B).

Additionally, the DMD reporter cell model was further analyzed using CLSM to visually assess the exon skipping efficiency. After treatment with 10 nM RNP, as described above, the cells were examined under a confocal microscope. The CLSM images (Figure 4C) revealed that in the single *1738* group, a limited proportion of cells displayed mCherry fluorescence, indicating exon skipping activity. In contrast, the *1738* plus Nu7441 group demonstrated a significantly higher proportion of mCherry-positive cells, aligning with the enhanced exon skipping efficiency observed in the flow cytometry results. In summary, Nu7441 enhanced NHEJ-mediated exon skipping in the DMD reporter cell model, consistent with its role in promoting NHEJ activity.

### 2.5. General Applicability of Nu7441 in Different Settings

**Figure 5:**
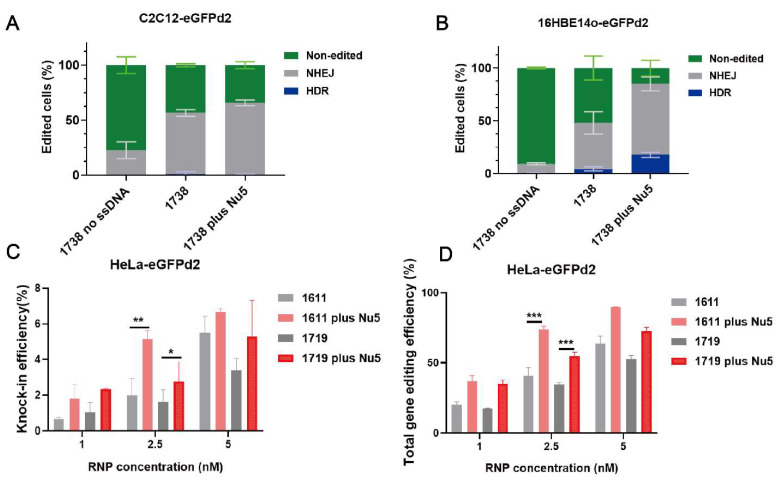
Gene correction and editing efficiency of Cas9 RNP/ssDNA complexes with NU7441 under various conditions. Gene editing was evaluated by flow cytometry in C2C12-eGFPd2 (**A**) and 16HBE14o-eGFPd2 (**B**) cell lines following transfection with *1738* Cas9 RNP complexes without a DNA template (referred to as *1738* no ssDNA, 18.75 nM) or *1738* Cas9 RNP/ssDNA complexes (18.75 nM) in the presence or absence of 5 nM Nu7441. The HDR efficiency (**C**) and total gene editing efficiency (**D**) of LAF Cas9 RNP/ssDNA complexes (*1611* and *1719*) with or without 5 nM Nu7441 in HeLa-eGFPd2. All complexes were prepared at a N/P ratio of 12 and transfected for 24 h. Data are shown as means ± SD (*n* = 3). Statistical significance is indicated as * *p* < 0.05, ** *p* < 0.01, and *** *p* < 0.001.

Nu7441 exhibited a high enhancement in gene editing, including HDR with the T-shaped lipo-XP-based Cas9 RNP/ssDNA system in the HeLa-eGFPd2 cell line. To further examine Nu7441’s role in HDR and gene editing under different conditions, we constructed C2C12-eGFPd2 and 16HBE14o-eGFPd2 cell lines. The cells were separately seeded and treated with the *1738*-based formulation under the same conditions as before (18.75 nM, RNP/ssDNA = 1/1), with or without 5 nM Nu7441. The results indicated that Nu7441 significantly improved the total gene editing efficiency in both the C2C12-eGFPd2 (Figure 5A, *p* = 0.0256) and 16HBE14o-eGFPd2 cell lines (Figure 5B, *p* = 0.0182). However, the HDR efficiency was only improved in the 16HBE14o-eGFPd2 cells, not in the C2C12-eGFPd2 cells. This could be attributed to cell-specific differences, such as variations in cell cycle dynamics or DNA repair machinery. Furthermore, we explored the impact of material structure on Nu7441’s performance. U-shaped lipo-amino fatty acid (LAF)-based XPs (*1611*, *1719*) [60,62,70] were formulated into Cas9 RNP/ssDNA complexes at a 1–5 nM RNP concentration and tested in HeLa-eGFPd2 cells. The results showed that Nu7441 continued to enhance the HDR and gene editing efficiency across structurally different delivery systems (Figure 5C,D). Similarly, Nu7441 strongly improved the HDR and total gene editing efficiency in HeLa-eGFPd2 cells using a Cas9 mRNA-based editing platform, specifically applying Cas9 mRNA/sgRNA/ssDNA polyplexes [60] formed with the LAF-XP carrier *1611* (Figure S5). This consistent enhancement highlights Nu7441 as a robust enhancer for both Cas9 protein/sgRNA/ssDNA and Cas9 mRNA/sgRNA/ssDNA delivery, making it a promising candidate for gene correction therapy.

### 2.6. Gene Correction Efficiency of Optimized T-Shaped Carrier 1636-Based Cas9 RNP/ssDNA Formulation with Nu7441

**Figure 6:**
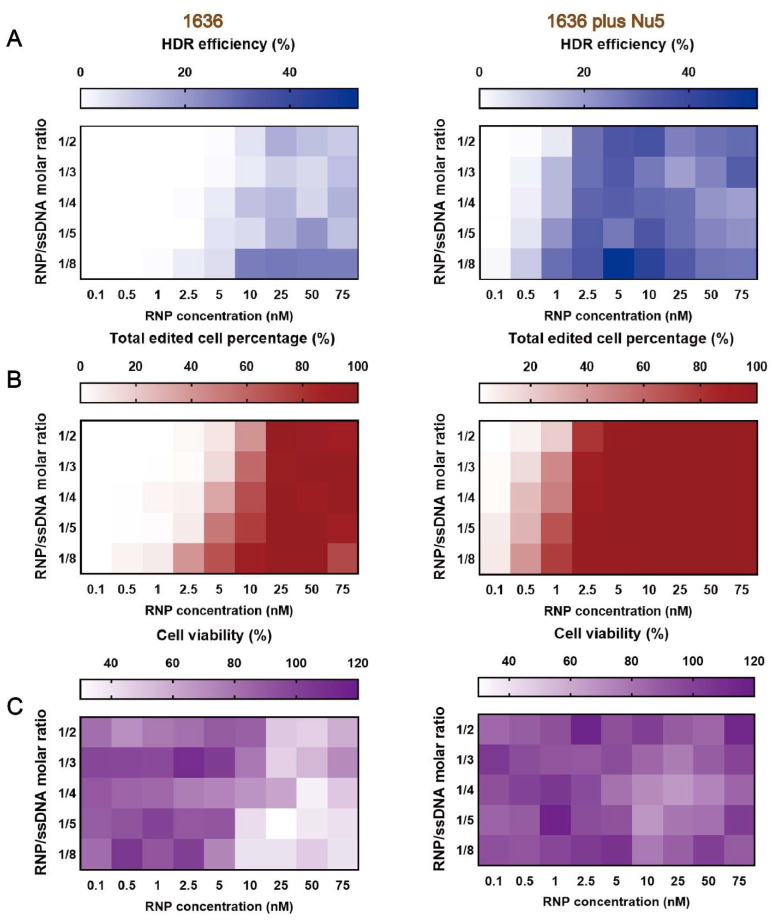
Enhanced HDR-mediated genome editing of HeLa-eGFPd2 applying the optimized HDR T-shaped XP (*1636*)-based Cas9 RNP/ssDNA complexes with Nu7441. (**A**) HDR efficiency, (**B**) total gene editing performance, and (**C**) cellular viability were evaluated after treatment with *1636*-based Cas9 RNP/ssDNA complexes at varying sgRNA/ssDNA ratios (1/2, 1/3, 1/4, 1/5, 1/8) and RNP concentrations ranging from 0.1 nM to 75 nM. Cytotoxicity was assessed using the MTT assay. All formulations maintained a N/P ratio of 12. Data are presented as the mean (*n* = 3). Numeric percentages are listed in Figure S6.

In our previous work, *1636* (Gtt) Cas9 RNP/ssDNA complexes achieved the highest HDR-mediated genome editing efficiency, reaching about 40% at a 25 nM RNP concentration and an RNP/ssDNA ratio of 1:4 after a 48-h transfection [39]. To evaluate the gene correction potential of Nu7441 in combination with these Cas9 RNP/ssDNA complexes, Cas9 RNP/ssDNA complexes were tested under similar conditions. HeLa-eGFPd2 cells were treated with the complexes, either with or without 5 nM Nu7441, for 24 h. Following treatment, the cells were incubated for an additional five days before analysis. The results demonstrated that the single *1636* group achieved over 26% HDR efficiency at a 25 nM RNP concentration and an RNP/ssDNA ratio of 1:8 (Figure 6A and Figure S6A). When supplemented with 5 nM Nu7441, the HDR efficiency was consistently improved in each group. For instance, at the lower RNP concentration of 5 nM and the same RNP/ssDNA ratio (1:8), the *1636* plus Nu7441 group achieved a remarkable HDR efficiency of 53% (Figure 6A and Figure S6A), highlighting Nu7441’s strong HDR enhancement effect.

A similar trend was observed for the total gene editing efficiency (Figure 6B and Figure S6B). Finally, MTT assays revealed improved cell metabolism in the *1636* plus Nu7441 group compared to the single *1636* group (Figure 6C and Figure S6C). This finding suggests that Nu7441 not only enhances the HDR efficiency but also reduces cytotoxicity associated with Cas9 RNP delivery, further supporting its potential as a versatile enhancer for genome correction and editing applications.

### 2.7. Comparison of HDR Efficiency in Flow Cytometry and Gene Sequence Measurement

**Figure 7:**
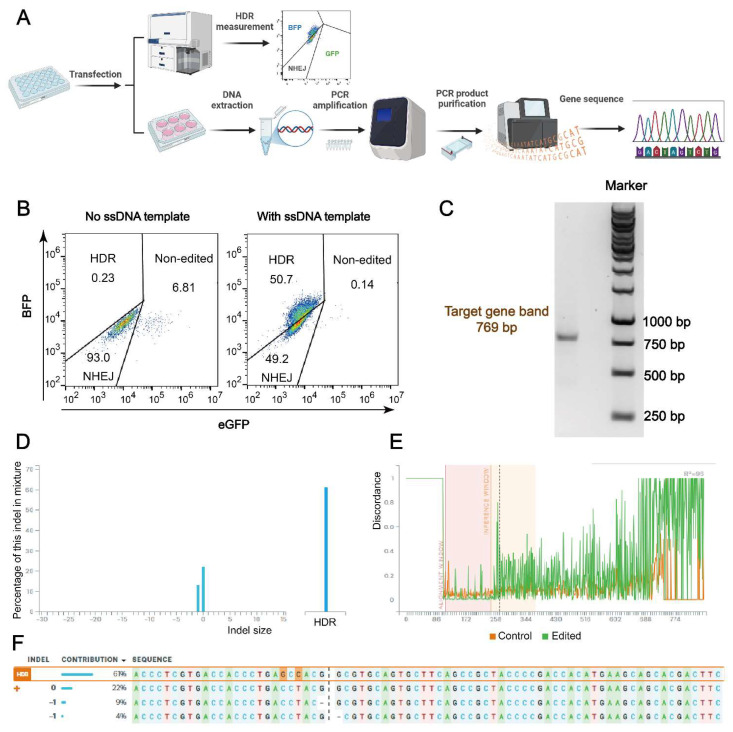
Gene sequence analysis of HeLa-eGFPd2 cells treated with Cas9 RNP/ssDNA complexes and Nu7441. (**A**) Schematic representation of the HDR evaluation process using flow cytometry and Sanger sequencing. (**B**) Flow cytometry gating strategy used to differentiate between eGFP-positive (non-edited), eGFP-negative (knock-out), and BFP-positive (knock-in) populations in samples treated with *1636* RNP/ssDNA complexes and 5 nM Nu7441. (**C**) Gel electrophoresis assay of PCR products (target gene sequence bands for GFP and BFP at 769 bp) from a sample exhibiting 50.7% HDR prior to Sanger sequencing. (**D**) Distribution of indel sizes. (**E**) Alignment of Sanger sequencing; control group is untreated HeLa-GFPd2 cells. (**F**) Contribution of each sequence after GFP-to-BFP conversion. Sanger sequencing results analyzed using the Synthego ICE tool (https://ice.synthego.com/#/, accessed on 18 October 2023).

HDR efficiency as measured by flow cytometry was further validated through gene sequence analysis. As depicted in Figure 7A, following the 24-h treatment and additional incubation, a portion of the HeLa-eGFPd2 cells was transferred to a six-well plate for continued incubation.

The cells were then harvested for DNA extraction. The extracted DNA was amplified using PCR, and the PCR products were purified before Sanger sequencing to confirm the HDR efficiency and validate the gene editing results. Figure 7B shows the gating strategy applied in all flow cytometry evaluations, indicating around 51% HDR efficiency. After the DNA extraction and PCR amplification of BFP in the sample shown in Figure 7B (50.7% HDR in FACS assay), a 769 bp target band was detected in an agarose gel electrophoresis experiment (Figure 7C). Next, the Sanger sequencing results compared with the untreated control cell group using the Synthego ICE tool, as described in previous studies [10,71], detected an HDR efficiency of 61% (Figure 7D–F), confirming the high-level gene sequence correction. This demonstrates that this platform could serve as a powerful tool for gene therapy. To further validate the gene sequence accuracy, purified HeLa-BFPd2 cells and an additional 50% sample were also analyzed, and the results confirmed the reliability of the findings (Figure S7). Additionally, a part of the samples was also detected using CLSM for further confirmation (Figure S8).

## 3. Discussion

DNA-PKcs pathway inhibitors, such as NU7026, Nu7441, and M3814, are known to effectively inhibit the NHEJ pathway, thereby enhancing the HDR efficiency. These inhibitors have been extensively studied in various cell models, including SW620, hiPSCs, K562, HEK293T, zebrafish embryos, and in CD34^+^ progenitor and CD4^+^ T cells, demonstrating a 3- to 13-fold improvement in the HDR efficiency [40]. For instance, NU7026 increased the targeted gene fragment insertion efficiency by 3-fold in HEK293 cells, 4-fold in K562 cells, 3-fold in CD4^+^ T cells, and 1.7-fold in CD34^+^ progenitor cells [48]. Similarly, M3814 enhanced the HDR efficiency to 81% by suppressing NHEJ in K562 cells [54], and Nu7441 improved the HDR efficiency by 3-fold in HEK293T cells [46] and by up to 13.4-fold in zebrafish embryos [72]. However, the effects of these inhibitors seem to be cell-type-dependent, as seen in our enhancer selection results (Figure 1), which may limit their broader application.

**Figure 8:**
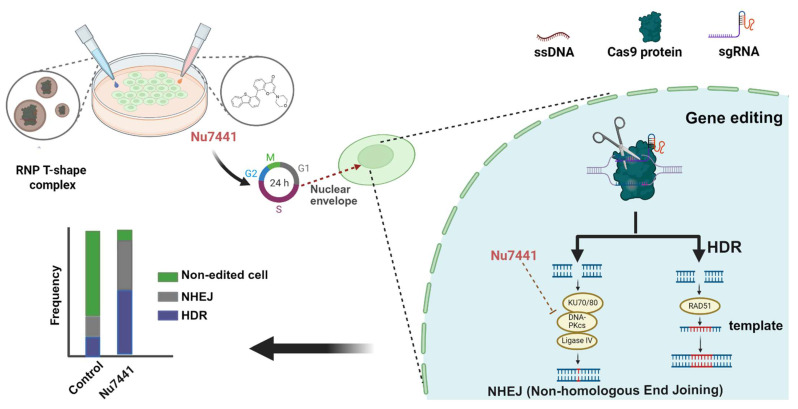
Schematic illustration of the gene editing process utilizing the lipo-xenopeptide-based RNP complex and the HDR enhancer Nu7441, with an additional possible impact on the cell cycle and the breakdown of the nuclear envelope.

In this study (see Figure 8), we developed a highly efficient gene correction and editing platform by combining lipo-XPs for CRISPR/Cas9 delivery with Nu7441. Our findings suggest that Nu7441 not only enhanced the HDR efficiency, as previously reported [46], but also improved the total gene editing efficiency, including NHEJ. Closer analysis revealed that the combination of Nu7441 with Cas9 RNP/ssDNA complexes did not affect the cellular uptake or endosomal escape of Cas9 RNP (Figure 3A,B); it did modulate the cell cycle by shifting more cells into the S and G2/M phases while reducing the G1 phase population (Figure 3E and Figure S2). Previous studies have shown that HDR is predominantly active in the S and G2 phases, which coincide with the availability of homologous sequences that can serve as repair templates during DNA damage [63,64]. Moreover, Jennifer A. Doudna’s group demonstrated that blocking the cell cycle at the M phase in HEK293T cells using nocodazole improved the HDR efficiency by up to 38% [73].

They speculated that delivering Cas9 RNP into nocodazole-synchronized cells might effectively target two daughter cells upon release from mitotic arrest. Another possibility is that the breakdown of the nuclear envelope during mitosis facilitates easier access for Cas9 RNP to the genomic DNA. Previous studies demonstrated that pDNA-mediated gene transfer is strongly promoted by transfection in the S or G2 phases close to the M phase, facilitated by nuclear membrane breakdown [65]. To generate indirect evidence, we co-transfected pDNA (GFP) and mRNA (mCherry) within the same nanoparticles in the presence or absence of Nu7441. According to our hypothesis, pDNA transfection (requiring nuclear entry) but not mRNA (requiring ribosomal translation in cytosol) should be enhanced by Nu7441. Indeed, we observed that Nu7441 enhanced pDNA transfection while reducing mRNA expression (Figure S3). Therefore, it is reasonable to hypothesize that Nu7441 enhances the HDR efficiency by arresting cells in the S and G2/M phases, thereby extending the DNA repair time in these phases and promoting Cas9/sgRNA nuclear entry. The mechanism likely ensures that more Cas9 reaches the nucleus at the optimal stage of the cell cycle and extends the repair time, which contributes to increased total gene editing efficiency, including HDR.

This modulation of the cell cycle also resulted in more efficient and specific gene knock-in or knock-out in HeLa, C2C12, and 16HBE14o- cells. Notably, gene correction efficiency improved by more than 10-fold in a GFP-to-BFP gene conversion model in HeLa cells and achieved over 50% at an RNP concentration of just 5 nM with the *1636*-based formulation, as measured by flow cytometry, with a corresponding 61% HDR efficiency observed in Sanger sequencing. Cas9-mediated exon skipping therapy has emerged as a promising approach for the treatment of Duchenne muscular dystrophy (DMD) [74]. In a DMD exon 23 reporter model, co-treatment with Nu7441 exhibited a 30-fold increase in the exon skipping efficiency, as shown in Figure 4 and Figure S4.

Although Nu7441 treatment reduced exogenous mRNA translation (Figure S3), the Cas9 mRNA/sgRNA/ssDNA polyplexes still exhibited higher HDR and gene editing efficiency when combined with Nu7441 (Figure S5). This suggests that Nu7441 provides a high potential for genomic DNA editing irrespective of the format of the gene editing cargo [23]. Moreover, gene correction and editing strategies based on NHEJ, transposon-mediated knock-in, prime editing (Cas nickases and reverse transcriptase), and base editing (Cas nickases and DNA deaminase) might also benefit from such effects. Therefore, we speculate that Nu7441 holds significant potential as a gene editing enhancer.

To quantify the editing outcomes, we primarily relied on the Synthego ICE tool for analyzing Sanger sequencing data. While ICE is widely used for indel quantification, it has known limitations, particularly in detecting subtle HDR edits involving 1–3 bp base changes. In our GFP-to-BFP conversion model, HDR introduces a precise point mutation that often produces minimal chromatogram shifts, which may be interpreted as “unedited” if the signal deviation falls below ICE’s detection threshold [75]. This limitation is particularly relevant when analyzing heterogeneous populations with mixed outcomes. For instance, while the flow cytometry in Figure 7B indicated 49.2% NHEJ and only 0.14% unedited cells, the ICE analysis in Figure 7F detected just 13% NHEJ and a higher unedited fraction (~22%). These discrepancies likely reflect the inherent limitations of the algorithm in accurately resolving subtle HDR events or low-frequency indels, as well as its reduced sensitivity to larger deletions and complex or mixed chromatogram signals. Moreover, Sanger sequencing itself may fail to capture complex editing patterns or deletions >30 bp due to read collapse in pooled samples [76]. Together, these considerations highlight the need to interpret ICE-based DNA-level analysis with caution in HDR-focused studies.

Another critical consideration is the potential for off-target effects. Although CRISPR/Cas systems are powerful tools, they are not without risk. Cas9 can cleave unintended genomic regions, particularly when up to three mismatches exist between the sgRNA and target DNA [77,78]. In silico tools are therefore essential for genome-wide off-target prediction [79]. In our study, NU7441 extends the DNA repair window and synchronizes cells in HDR-permissive phases (S and G2/M). While this enhances on-target HDR (Figure 7), it may also inadvertently increase susceptibility to off-target events by prolonging Cas9 activity or enabling unintended integration during extended repair periods. These findings underscore the importance of further evaluating the off-target profile of this approach, especially when applying it to therapeutic contexts.

In parallel with concerns about off-target genome editing, the safety profile of NU7441 must also be carefully considered. Although NU7441 remains a preclinical compound and has not yet entered clinical trials, several studies have reported its potential side effects based on animal models and combination treatments in cancer research. Zhao et al. reported that NU7441 exhibited high hepatic accumulation, raising concerns about potential hepatotoxicity [80]. Moreover, its poor aqueous solubility may pose formulation challenges [81]. When used in combination with chemotherapy agents (e.g., etoposide) [82] or radiation [83], NU7441 has been shown to enhance the therapeutic effect by sensitizing cancer cells to DNA damage. However, this radiosensitization or chemosensitization effect may also exacerbate systemic toxicity, potentially increasing side effects such as nausea, vomiting, diarrhea, and fatigue.

Additional limitations of our current study should also be acknowledged. First, our experiments were primarily performed using a limited set of target sites and gene sizes. Thus, the generalizability of Nu7441’s effect across different genomic loci, especially larger or clinically relevant genes, remains to be evaluated. Second, although we expanded the scope beyond cancer cell lines by including non-transformed 16HBE14o- and C2C12-cells, we did not extend the validation to a broader range of tissue types or primary cells lacking functional reporters. In particular, assessing genome editing at endogenous loci across diverse cell types—including patient-derived primary cells or iPSC-derived models—would be critical for evaluating the therapeutic potential in more physiologically relevant contexts. Third, a more comprehensive evaluation of potential off-target effects was beyond the scope of this study. Future work involving genome-wide off-target profiling (e.g., GUIDE-seq or DISCOVER-seq) [84,85] and the application of our editing strategy in primary or disease-relevant cell models will be essential for advancing the translational relevance of these findings.

In conclusion, this study presents a highly efficient gene editing platform combining most recently developed lipo-XPs for CRISPR/Cas9 delivery with Nu7441, a DNA-PKcs pathway inhibitor. The platform enhances the HDR efficiency and total gene editing by modulating the cell cycle, specifically by extending the DNA repair time in the S and G2 phases and promoting Cas9 RNP nuclear entry upon mitosis in the M phase. This strategy results in efficient gene knock-in or knock-out across various cell models and non-viral delivery systems.

## 4. Materials and Methods

### 4.1. Materials

Nu7441 (KU-57788), Rs1, KU-0060648, Scr7, Pevonedistat (MLN4924), and M3814 (nedisertib) were purchased from MedChemExpress (Monmouth Junction, NJ, USA). Reagents including 4′,6-diamidino-2-phenylindole (DAPI), propidium iodide (PI), ampicillin, dimethyl sulfoxide (water-free) (DMSO), Dulbecco’s modified Eagle’s medium (DMEM), fetal bovine serum (FBS), penicillin/streptomycin, and 3-(4,5-dimethylthiazol-2-yl)-2,5-diphenyltetrazolium bromide (MTT) were obtained from Life Technologies (Carlsbad, CA, USA). Minimum essential medium (MEM) and Lipo 3k were procured from Thermo Fisher Scientific (Waltham, MA, USA), and 4-(2-hydroxyethyl)-1-piperazineethanesulfonic acid (HEPES) was sourced from Biomol GmbH (Hamburg, Germany). HEPES-buffered glucose solution (HBG, pH 7.4) was prepared by dissolving 20 mM HEPES and 5% (*w*/*v*) glucose in water. Cas9 protein was harvested and labeled with ATTO647N, as previously described [38,39]. Cas9 mRNA (mCherry) was obtained from Trilink Biotechnologies (San Diego, CA, USA). Single guide RNA (sgRNA) with 2′-O-methyl modifications on the first and last three RNA bases, along with phosphorothioate modifications on the RNA bases, Cas9 mRNA, and single-stranded DNA (ssDNA) were procured from Integrated DNA Technologies (Coralville, IA, USA) or AxoLabs GmbH (Kulmbach, Germany), with sequences described in prior studies [60,70]. pEGFP-N1 plasmid was obtained from addgene (https://www.addgene.org/178088/, accessed on 25 August 2022).

Deionized water, purified using a Millipore system (Simplicity Plus, Millipore Corp Burlington, MA, USA), was employed for solution preparation. The synthesis of the artificial amino acid Fmoc-Stp(Boc)_3_-OH (Stp) and its analogues was performed as previously described [39,60].

The lipo-XPs *1738* (dGtp) and *1636* (Gtt) were synthesized using standard Fmoc solid-phase synthesis and detected by MALDI, as previously described [18]. Sequences of all tested lipo-XPs are listed in Tables S2 and S3. The synthesized oligomers were stored at a concentration of 10 mg/mL at −20 °C.

### 4.2. Fabrication of Cas9 Ribonucleoprotein (RNP) Complexes or mRNA/sgRNA Polyplexes

To prepare the Cas9 RNP/ssDNA complex, pre-stored Cas9 protein and sgRNA solution were mixed and incubated for 15 min at room temperature. The resulting Cas9 RNP solution was then diluted with HBG buffer (pH 7.4). Subsequently, an equal volume of ssDNA template solution was added to the diluted Cas9 RNP solution and thoroughly mixed by pipetting 10 times. The mixture was further combined with an equal volume of the oligomer solution and incubated at room temperature for 40 min.

For the preparation of the Cas9 RNP complex (without ssDNA), the pre-mixed Cas9 RNP solution was directly added to the oligomer solution at an equal volume, followed by incubation at room temperature for 15 min.

The final Cas9 RNP/ssDNA complexes were prepared at concentrations of 100 nM or 75 nM with a nitrogen-to-phosphate (N/P) ratio of 12, while the Cas9 RNP complexes were formulated with a N/P ratio of 24. These formulations were then used in subsequent experiments.

Cas9 mRNA nanoparticles were prepared by diluting Cas9 mRNA, sgRNA, and ssDNA at a weight ratio of 1:1:1.2 in HBG. The final concentration of nucleic acid in HBG was 25 ng/µL. The lipo-XP 1611 was diluted in water to a final concentration of 0.4 mg/mL. Equal amounts of nucleic acid dilution and lipo-XP dilution were rapidly mixed, and the obtained polyplexes were incubated for 40 min at room temperature.

### 4.3. Characterization of Cas9 RNP/ssDNA Complexes

The size and zeta potential of the Cas9 RNP/ssDNA complexes were determined using a Zetasizer Nano ZS (Malvern Instruments, Worcestershire, UK) under the following conditions: equilibration time of 30 s, temperature of 25 °C, refractive index of 1.330, and viscosity of 0.8872 mPa·s. Additionally, the morphology of the Cas9 RNP/ssDNA complex was analyzed using transmission electron microscopy (TEM).

For TEM sample preparation, the grid was positioned with its activated side facing a 10 μL droplet of the sample. Excess liquid was gently removed using filter paper. The staining process was performed in two steps: First, the grid was rinsed with 5 μL of 1.0% uranyl formate solution, which was immediately removed. Then, another 5 μL of the same staining solution was applied to the grid. After the excess solution was blotted away with filter paper, the grid was allowed to air-dry for 15 min. The samples were then analyzed using a JEOL JEM-1100 electron microscope (Tokyo, Japan) operated at an acceleration voltage of 80 kV.

### 4.4. GFP-to-BFP Conversion Mediated via Homology-Directed Repair (HDR)

HeLa-eGFPd2, C2C12-eGFPd2, and 16HBE14o-eGFPd2 cells were seeded individually into Corning^®^ Costar 96-well plates one day prior to the experiment. HeLa-eGFPd2 and C2C12-eGFPd2 cells were plated at a density of 5 × 10^3^ cells per well, while 16HBE14o-eGFPd2 cells were seeded at a density of 1 × 10^4^ cells per well. After 24 h of cell attachment, Cas9 RNP/ssDNA complexes or Cas9 mRNA/sgRNA/ssDNA polyplexes, prepared at specified concentrations optimized for each cell line, were added to each well containing fresh culture medium. Simultaneously, 10 μL of various enhancers, pre-diluted in medium to the desired working concentrations, was added to the wells. The resulting mixtures were incubated with the cells at 37 °C for 24 h under standard culture conditions.

The cells were collected by trypsinization, centrifuged at 1000× *g* for 5 min, washed with PBS, and resuspended in FACS buffer (10% FBS in PBS, *v*/*v*). Before measurement, 1 μg/mL propidium iodide (PI) (Thermo Fisher Scientific, Waltham, MA, USA) was added to distinguish viable cells from dead cells. GFP-positive (unedited), GFP-negative (NHEJ), and BFP-positive (HDR) cells were quantified from 3000 viable cells using a Beckman Coulter CytoFLEX S flow cytometer (Carlsbad, CA, USA). PI, BFP, and GFP signals were detected with excitation/emission wavelengths of 561/610 nm, 405/450 nm, and 488/530 nm, respectively.

### 4.5. Cellular Uptake and Endosomal Escape of Cas9 RNP/ssDNA Complexes

In the case of the cellular uptake, HeLa cells were seeded at a density of 2.5 × 10^4^ cells per well in 24-well plates and allowed to grow overnight prior to the experiment. The following day, the cells were incubated with *1738*-based Cas9 RNP/ssDNA complexes (37.5 nM, including 20% ATTO647N-labeled Cas9 protein) in the presence or absence of 50 nM Nu7441 for 2 h. To remove any nanoparticles bound to the cell membrane, the cells were rinsed with 500 μL of PBS containing 2000 IU heparin (Sigma-Aldrich, Merck, Darmstadt, Germany). Subsequently, they were kept on ice for 30 min. The cells were then washed with PBS to prepare for nuclear staining with DAPI (Thermo Fisher Scientific, Waltham, MA, USA). Afterward, they were harvested and analyzed using a Beckman Coulter CytoFLEX S flow cytometer. Each experiment was performed in triplicate. Fluorescence detection was carried out with excitation at 405 nm and emission at 450 nm for DAPI and excitation at 640 nm with emission at 670 nm for ATTO647N.

For the endosomal escape experiment, HeLa Gal8-mRuby3 cells were placed at a density of 2 × 10^4^ cells per well in 8-well Ibidi µ-slides (Ibidi GmbH, Gräfelfing, Germany) and incubated for 24 h prior to transfection. Subsequently, 280 µL new medium, 80 µL of RNP complexes, and 40 µL of Nu7441 (50 nM) were added to each well, followed by an incubation period of 2 h. Cells were further washed with 300 μL PBS, fixed with 4% paraformaldehyde (Sigma-Aldrich, Merck, Darmstadt, Germany) at room temperature for 45 min, and stained with 1 µg/mL DAPI in the dark for 15 min. After replacing the staining solution with 300 μL fresh PBS, the samples were analyzed using a Leica TCS SP8 confocal microscope (Leica Microsystems, Wetzlar, Germany). DAPI signals were recorded at 450 nm and mRuby3 signals at 590 nm. Gal8 spots per cell were quantified using ImageJ (version 1.48, National Institutes of Health, Bethesda, MD, USA) by removing nuclei and background, converting the image to 8 bit, adjusting thresholds, and counting cytosolic spots with the Analyze Particles tool.

### 4.6. Cellular Cell Cycle Assay

HeLa cells were seeded at a density of 1.2 × 10^4^ cells per well in Corning^®^ Costar 96-well plates (Corning Inc., Corning, NY, USA) one day prior to treatment. For the pre-Nu5 group, cells were pre-incubated with 5 nM Nu7441 for 24 h on the first day. After incubation, the cells were washed with PBS and treated with Cas9 RNP/ssDNA complexes on the second day for an additional 24 h. For the co-Nu5 group, cells were simultaneously treated with Cas9 RNP/ssDNA complexes and 5 nM Nu7441 for 24 h on the second day. The single *1738* group (control) was treated with Cas9 RNP/ssDNA complexes alone under the same conditions on the second day. For the normal transfection protocol in HeLa-GFPd2, the cells were directly incubated with the *1738* RNP complex/ssDNA and 5 nM Nu7441, as described above. On the last day, cells from all groups were collected and suspended dropwise into 90 µL of cold 70% ethanol. The suspensions were incubated at 4 °C for 2 h. Subsequently, the cells were centrifuged and resuspended in 100 µL of PI staining buffer (comprising 0.1% sodium citrate, 0.1% Triton X-100, 180 U/mL RNase stock solution, and 50 µg/mL PI). The suspensions were incubated for 3 h at 4 °C. Finally, the cells were washed with PBS and analyzed using flow cytometry.

### 4.7. Gene Editing-Mediated Exon Skipping of Cas9 RNP Complexes with or Without Nu7441

HeLa mCherry-DMD_Ex23_ cells were plated at a density of 5 × 10^3^ cells per well in Corning^®^ Costar 96-well plates and maintained for 24 h before treatment. Cas9 RNP complexes (20 μL) formed with sgDMDEx23 as described in prior studies [60,70] with or without Nu7441 (10 μL) were then applied to the cells for 24 h. Prior to transfection, nanocarriers were assembled as previously detailed and diluted with HBG to achieve the desired concentration in the final 20 μL solution. Following the transfection period, cells were further cultured for two days. Subsequently, the cells were detached using trypsin, centrifuged at 1000× *g* for 5 min, and rinsed with PBS. The collected cells were resuspended in FACS buffer and analyzed for gene editing efficiency using a Beckman Coulter CytoFLEX S flow cytometer according to the previously described protocol. DAPI and mCherry signals were recorded at 450 nm and 610 nm, respectively.

### 4.8. Gene Editing-Mediated Exon Skipping by Cas9 RNP Complexes Visualized by CLSM

HeLa mCherry-DMD_Ex23_ cells were seeded at 2 × 10^4^ cells per well in Corning^®^ Costar 24-well plates and cultured for 24 h. The cells were then transfected with 10 nM Cas9 RNP complexes, with or without 5 nM Nu7441, for 24 h, followed by two additional days of incubation. Afterward, the cells were transferred to 8-well Ibidi µ-slides and incubated overnight. The following day, cells were washed with 300 μL PBS, fixed with 4% paraformaldehyde for 45 min, and stained for 2 h with Alexa Fluor™ 488 phalloidin (Thermo Fisher Scientific, Waltham, MA, USA) to label the cytoskeleton. Nuclei were stained with 1 µg/mL DAPI in the dark for 15 min. After staining, the solution was replaced with fresh PBS. Samples were then analyzed using a Leica TCS SP8 confocal microscope. DAPI, Alexa Fluor™ 488, and mCherry signals were recorded at 450 nm, 530 nm, and 610 nm, respectively.

### 4.9. Genome Extraction

The treated HeLa eGFPd2 cells underwent the “GFP-to-BFP conversion mediated via homology-directed repair (HDR)” process as described. The cells were transferred into 24- and 6-well plates to promote growth expansion. After sufficient growth, the cells were harvested, and DNA was extracted using the QIAamp DNA Mini Kit (Hilden, Germany). The cells were washed with PBS and resuspended in 100 µL of PBS. Subsequently, 10 µL of proteinase K and 100 µL of buffer AL were added to the cell suspension. The mixture was incubated at 56 °C for 10 min. Following incubation, 100 µL of ethanol was added to the sample, which was vortexed for 15 s. The mixture was then transferred into a spin 2.0 column and centrifuged at 8000× *g* for 1 min. The liquid from the collection tube was discarded, and 500 µL of buffer AW1 was added to the column, followed by centrifugation at 8000× *g* for 1 min. The sample was subsequently washed with 500 µL of buffer AW2 and centrifuged at 13,300× *g* for 3 min. The waste buffer was discarded, and the column was centrifuged empty for an additional 1 min to ensure complete removal of residual buffer. Finally, 50 µL of nuclease-free water was added to the column, and centrifugation was performed to obtain the purified DNA sample.

### 4.10. PCR Amplification and Sequencing

To obtain the target gene sequence for gene sequence measurement, primers (TGGGCAACGTGCTGGTTATT, CACGAACTCCAGCAGGACCATG) were designed for PCR amplification. The harvested DNA sample (100 ng) was mixed with 10× Thermopol standard buffer, 10 mM dNTPs, 10 µM forward/reverse primer, nuclease-free water, and Taq DNA polymerase (New England Biolabs, Ipswich, Massachussets, USA). The mixture was added to a PCR tube and subjected to the following PCR conditions: initial denaturation (94 °C, 30 s), 37 cycles (94 °C, 30 s; 58 °C, 1 min), and final extension (68 °C, 1 min).

Subsequently, a 2% (*w*/*v*) agarose gel was prepared by dissolving agarose (Sigma-Aldrich, Merck, Darmstadt, Germany) in TBE buffer (containing 18.0 g tris(hydroxymethyl)aminomethane, 5.5 g boric acid, and 0.002 M disodium ethylenediaminetetraacetic acid (EDTA) at pH 8, in 1 L of water). After cooling to approximately 50 °C, 1× GelRed™ (Hayward, CA, USA) was added. The agarose solution was cast into an electrophoresis unit and allowed to solidify. For the PCR products, 5 μL of 6× loading buffer (6 mL glycerol, 1.2 mL 0.5 M EDTA, 2.8 mL water, and 0.02 g bromophenol blue) was added to 25 μL of PCR product. Samples were loaded into the gel wells, and electrophoresis was performed at 100 V for 3 h in TBE buffer. The unedited cell PCR products served as a control. The peqGold 1 kb DNA Ladder (VWR International, Radnor, PA, USA) was used to estimate DNA fragment sizes and confirm the accuracy of PCR amplification and cloning.

The full-length band site of 769 bp was identified by comparing it with the marker. The targeted band was excised using a scalpel, and the PCR products were purified using a QIAquick PCR purification kit (Hilden, Germany) before being sent to Eurofins for Sanger sequencing. Final gene sequence results were analyzed using the Synthego ICE tool (https://ice.synthego.com/#/, accessed on 18 October 2023).

### 4.11. Assessment of Cytotoxicity of Cells via MTT Assay

Following 24 or 48 h of transfection, as described above, 10 μL of MTT solution was added to achieve a final concentration of 0.5 mg/mL. After 3 h of incubation at 37 °C, the supernatant was discarded, and the plates were stored at −80 °C for at least 1 h. Formazan crystals were dissolved in 100 μL of DMSO and incubated at 37 °C for 30 min with shaking at 125 rpm. Absorbance was recorded at 590 nm with a background correction at 630 nm using a Tecan Spectrafluor Plus microplate reader (Tecan, Männedorf, Switzerland). Cell viability (%) was calculated as (*A*/*B*) × 100, where *A* and *B* represent the absorbance of the test sample and HBG group, respectively.

### 4.12. Statistical Analysis

Statistical analysis was performed using GraphPad Prism 8.0 software, with the results presented as the mean ± standard deviation (SD). Data were evaluated through an unpaired, two-tailed Student’s *t*-test, where statistical significance was denoted as * *p* < 0.05, ** *p* < 0.01, *** *p* < 0.001, and **** *p* < 0.0001, with “ns” representing no significance.

## Data Availability

Data are contained within this article and the Supplementary Materials.

## Supplementary Materials

The following supporting information can be downloaded at https://www.mdpi.com/article/10.3390/ijms26094361/s1. Refs [38,59,69,86] are cited in Supplementary Materials file.

## Abbreviations

The following abbreviations are used in this manuscript:

Cas9	CRISPR-associated protein 9
chGtp	Cyclohexyl-glutaryl-tetraethylene pentamine
CLSM	Confocal laser scanning microscopy
CRISPR	Clustered regularly interspaced short palindromic repeat
DAPI	4′,6-Diamidino-2-phenylindole
DSBs	Double-strand breaks
DNA-PKcs	DNA-dependent protein kinase
dGtp	Diglycoloyl tetraethylene pentamine
DLS	Dynamic light scattering
DMD	Duchenne muscular dystrophy
DMEM	Dulbecco’s modified Eagle’s medium
DMSO	Dimethyl sulfoxide
FACS	Flow cytometry staining
FBS	Fetal bovine serum
LAF	Lipo-amino fatty acid
GEIPA	Glutaryl-ethylenediImine-dipropylamine
Gtt	Glutaryl-triethylene tetramine
HBG	HEPES-buffered glucose solution
HEPES	4-(2-Hydroxyethyl)-1-piperazineethanesulfonic acid
HDR	Homology-directed repair
N/P	Nitrogen-to-phosphate
NHEJ	Non-homologous end joining
NLRP3	NOD-like receptor family pyrin domain containing 3
MEM	Minimum essential medium
MFI	Median fluorescence intensity
MTT	3-(4,5-Dimethylthiazol-2-yl)-2,5-diphenyltetrazolium bromide
sgRNA	Single guide RNA
ssDNA	Single-stranded DNA
PD-L1	Programmed cell death ligand 1
PI	Propidium iodide
RNP	Ribonucleoprotein
Stp	Succinyl-tetraethylene pentamine
TEM	Transmission electron microscopy
TFE	Trifluoroethyl-iminodiacetyl-tetraethylene pentamine
XP	Xenopeptide
