# Supplementary material for: CRISPR/Cas9 Ribonucleoprotein Delivery Enhanced by Lipo-Xenopeptide Carriers and Homology-Directed Repair Modulators: Insights from Reporter Cell Lines
Source: Int J Mol Sci. 2025 May 3;26(9):4361. doi: 10.3390/ijms26094361 (PMC12073011; doi:10.3390/ijms26094361)
Supplement: Supplementary file 1 [file ijms-26-04361-s001.zip › ijms-3556183-supplementary.pdf]

Supplementary Materials

Table S1 The sequences of the sgRNA, ssDNA and GFPd2 gene sequence used for GFP conversion, as well as the sgRNA and mcherry-DMDEX 23 gene sequence for exon skipping in this study.

Name	Sequence 5'→ 3'
sgGFP	5'<br>mG*mC*mU*GAAGCACUGCACGCCGUGUUUUAGAGCUAGAAAUAGCAAGUUAAAAUAAGGCUAGU<br>CCGUUAUCAACUUGAAAAAGUGGCACCGAGUCGGUGCmU*mU*mU*U 3'
ssDNA	5'<br>G*C*CACCTACGGCAAGCTGACCCTGAAGTTCATCTGCACCACCGGCAAGCTGCCCGTGCCCTGGCCCAC<br>CCTCGTGACCACCCTGAGCCACGGCGTGCAGTGCTTCAGCCGCTACCCCGACCACAT*G*A 3'
sgDMDEx23	5'<br>mA*mU*mU*UCAGGUAAGCCGAGGUUGUUUUAGAGCUAGAAAUAGCAAGUUAAAAUAAGGCUAG<br>UCCGUUAUCAACUUGAAAAAGUGGCACCGAGUCGGUGCmU*mU*mU*U 3'
GFPd2 gene sequence	5'<br>ATGGTGAGCAAGGGCGAGGAGCTGTTCACCGGGGTGGTGCCCATCCTGGTCGAGCTGGACGGCGACGTA<br>AACGGCCACAAGTTCAGCGTGTCCGGCGAGGGCGAGGGCGATGCCACCTACGGCAAGCTGACCCTGAAG<br>TTCATCTGCACCACCGGCAAGCTGCCCGTGCCCTGGCCCACCCTCGTGACCACCCTGACCTACGGCGTGC<br>AGTGCTTCAGCCGCTACCCCGACCACATGAAGCAGCACGACTTCTTCAAGTCCGCCATGCCCGAAGGCTA<br>CGTCCAGGAGCGCACCATCTTCTTCAAGGACGACGGCAACTACAAGACCCGCGCCGAGGTGAAGTTCGA<br>GGGCGACACCCTGGTGAACCGCATCGAGCTGAAGGGCATCGACTTCAAGGAGGACGGCAACATCCTGGG<br>GCACAAGCTGGAGTACAACTACAACAGCCACAACGTCTATATCATGGCCGACAAGCAGAAGAACGGCATC<br>AAGGTGAACTTCAAGATCCGCCACAACATCGAGGACGGCAGCGTGCAGCTCGCCGACCACTACCAGCAG<br>AACACCCCCATCGGCGACGGCCCCGTGCTGCTGCCCGACAACCACTACCTGAGCACCCAGTCCGCCCTGA<br>GCAAAGACCCCAACGAGAAGCGCGATCACATGGTCCTGCTGGAGTTCGTGACCGCCGCCGGGATCACTC<br>TCGGCATGGACGAGCTGTACAAGAAGCTTAGCCATGGCTTCCCGCCGGAGGTGGAGGAGCAGGATGATG<br>GCACGCTGCCCATGTCTTGTGCCCAGGAGAGCGGGATGGACCGTCACCCTGCAGCCTGTGCTTCTGCTAG<br>GATCAATGTGTAG3'
mCherry-DMDEx23 gene sequence	5'<br>ATGGAGGGCTCCGTGAACGGCCACGAGTTCGAGATCGAGGTAAGGGCACTGAGCAGAAGGGAAGAAGC<br>TCCGGGGGCTCTTTGTAGGGTCCTCCAGTCAGGACTCAAACCCAGTAGTGTCTGGTTCCAGGCACTGACC<br>TTGTATGTCTCCTGGCCCAAATGCCCACTCAGGGTAGGGGTGTAGGGCAGACAACGAGTCTTTTGTCATC<br>TACAGGCTCTGCAAAGTTCTTTGAAAGAGCAATAAAATGGCTTCAACTATCTGAGTGACACTGTGAAGGA<br>GATGGCCAAGAAAGCACCTTCAGAAATATGCCAGAAATATCTGTCAGAATTTGAAGAGATTGAGGGGCAC<br>TGGAAGAAACTTTCCTCCCAGTTGGTGGAAAGCTGCCAAAAGCTAGAAGAACATATGAATAAACTTCGAA<br>AATTTCAGGTAAGCCGAGGTTTGGCCTTGGAAGAAGCTCCGGGGGCTCTTTGTAGGGTCCTCCAGTCAGG<br>ACTCAAACCCAGTAGTGTCTGGTTCCAGGCACTGACCTTGTATGTCTCCTGGCCCAAATGCCCACTCAGG<br>GTAGGGGTGTAGGGCAGACAACGAGTCTTTTGTCATCTACAGGGCGAGGGCGAGGGCCGCCCCTACGAG<br>GGCACCCAGACCGCCAAGCTGAAGGTGACCAAGGGTGGCCCCCTGCCCTTCGCCTGGGACATCCTGTCC<br>CCTCAGTTCATGTACGGCTCCAAGGCCTACGTGAAGCACCCCGCCGACATCCCCGACTACTTGAAGCTGT<br>CCTTCCCCGAGGGCTTCAAGTGGGAGCGCGTGATGAACTTCGAGGACGGCGGCGTGGTGACCGTGACCC<br>AGGACTCCTCCCTGCAGGACGGCGAGTTCATCTACAAGGTGAAGCTGCGCGGCACCAACTTCCCCTCCGA<br>CGGCCCCGTAATGCAGAAGAAGACCATGGGCTGGGAGGCCTCCTCCGAGCGGATGTACCCCGAGGACGG<br>CGCCCTGAAGGGCGAGATCAAGCAGAGGCTGAAGCTGAAGGACGGCGGCCACTACGACGCTGAGGTCA<br>AGACCACCTACAAGGCCAAGAAGCCCGTGCAGCTGCCCGGCGCCTACAACGTCAACATCAAGTTGGACAT<br>CACCTCCCACAACGAGGACTACACCATCGTGGAACAGTACGAACGCGCCGAGGGCCGCCACTCCACCGG<br>CGGCATGGACGAGCTGTACAAGTAAGGATCC3'

Modification pattern: '*' denotes phosphorothioated RNA bases; 'm' indicates 2'-O-methyl nucleotide bases.

Table S2 The xenopeptide (XP) library in this study, consisting of *1392*-, *1396*-, and *1445*-analogues, includes IDs and sequences (N- to C-terminus).

Backbone	ID	Sequence
1392 analogues	1392	K(N_3)-Y_3-Stp-K(K(LinA)$_2$)-Stp-Y_3
	1737	K(N_3)-Y_3-chGtp-K(K-(LinA)$_2$)-chGtp-Y_3
	1738	K(N_3)-Y_3-dGtp-K(K-(LinA)$_2$)-dGtp-Y_3
1396 analogues	1396	K(N_3)-Y_3-H-Stp-H-K(K(LinA)$_2$)-H-Stp-H-Y_3
	1653	K(N_3)-Y_3-H-GEIPA-H-K(K(LinA)$_2$)-H-GEIPA-H-Y_3
	1743	K(N_3)-Y_3-H-TFE-H-K(K(LinA)$_2$)-H-TFE-H-Y_3
1445 analogues	1445	K(N_3)-C-Y_6-Stp_2-K(K-(OleA)$_2$)-Stp_2-Y_6-C
	1636	K(N_3)-C-Y_3-Gtt_2-K(K(OHSteA)$_2$)-Gtt_2-Y_3-C
	1637	K(N_3)-C-Y_3-$GEIPA_2$-K(K(OHSteA)$_2$)-$GEIPA_2$-Y_3-C

Table S3 The XP ID and list of sequences (N- to C-terminus) of *1218* and LAF-XPs *(1611* and *1719)*.

ID	Sequence
1218	K(N_3)-Y_3-(H-Stp)$_2$-H-K(G-ssbb-K(OleA)$_2$)-H-(Stp-H)$_2$-Y_3
1611	(12Oc)K-Stp-K(12Oc)
1719	[K(12Oc)]$_2$-Stp_2-[K(12Oc)]$_2$

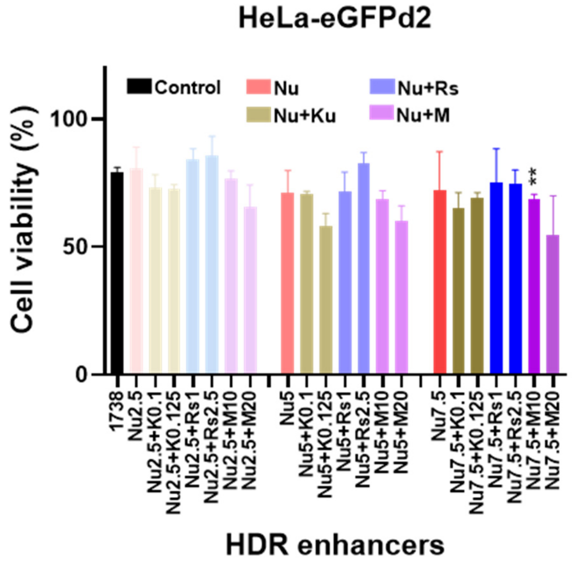

Figure S1. Cell viability of HeLa-eGFPd2 post-transfection with 1738 Cas9 RNP/ssDNA complexes and enhancer mixtures. Cell viability was assessed for 18.75 nM *1738* Cas9 RNP/ssDNA complexes, with or without enhancer mixtures, 24 h post-transfection. The enhancer mixtures included Nu7441 (2.5, 5, 7.5 nM), Rs1 (1, 2.5, 10 nM), KU-0060648 (0.1, 0.25, 1 nM), or M3814 (5, 10, 20 nM). Enhancer abbreviations represent the enhancer name followed by its concentration in nM. The experiment was performed using the MTT assay. ** $p < 0.01$ vs *1738*; Data are presented as mean ± SD ($n = 3$).

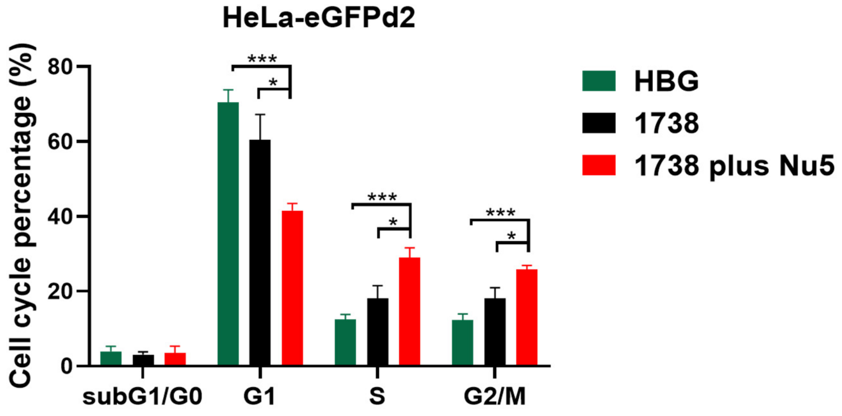

Figure S2. Cell cycle analysis of HeLa-GFPd2 cells post-transfection with 1738 RNP/ssDNA complex and Nu7441. The analysis was performed using PI staining following a 24-hour transfection with *1738*-based RNP/ssDNA complex (18.75 nM RNP) with or without 5 nM Nu7441 and an additional 5-day incubation in HeLa-GFPd2 cells. Data are presented as mean ± SD ($n = 3$). Statistical significance is indicated as * $p < 0.05$ and *** $p < 0.001$.

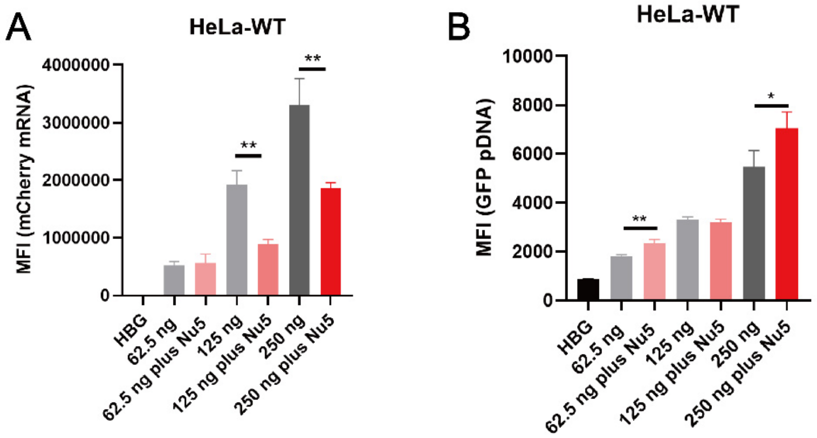

Figure S3. Median Fluorescence Intensity (MFI) analysis of mRNA and DNA co-transfection in HeLa cells with or without Nu7441. The analysis of mRNA and DNA co-transfection in HeLa cells without or with Nu7441. (**A**) mRNA (mCherry) and (**B**) pDNA (GFP) MFI results after 24 h of treatment with *1611*-based mRNA/pDNA polyplexes, in the presence or absence of 5 nM Nu7441. The polyplexes were formed at an N/P ratio of 18 and contained a total of 62.5 ng, 125 ng, or 250 ng of nucleic acid (mCherry mRNA: GFP pDNA; weight ratio 1:1). Data are presented as mean ± SD ($n = 3$). Statistical significance is indicated as * $p < 0.05$ and ** $p < 0.01$.

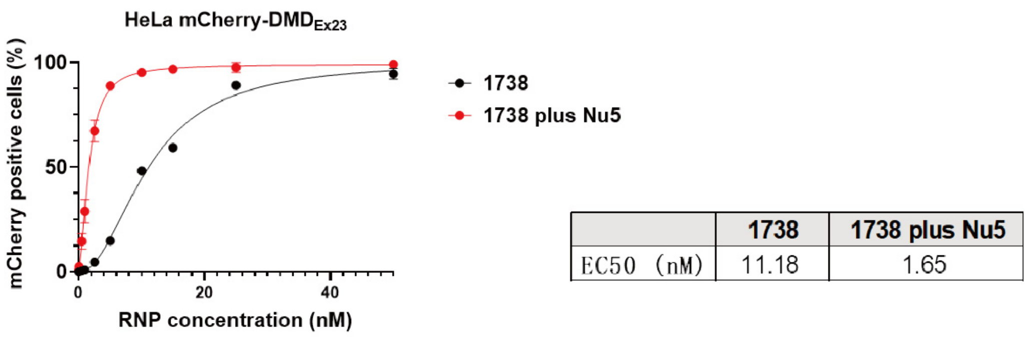

	1738	1738 plus Nu5
EC50 (nM)	11.18	1.65

Figure S4. Exon skipping dose titration of *1738* and *1738* plus Nu5 in HeLa mCherry-DMD$_{Ex23}$. Cas9 RNP concentrations ranged from 0.05 to 50 nM. EC50 value calculated as dose for 50% mCherry positive cells. Addition of 5 nM Nu7441 reduced EC50 by 6.8 fold. Data are presented as mean ± SD ($n = 3$).

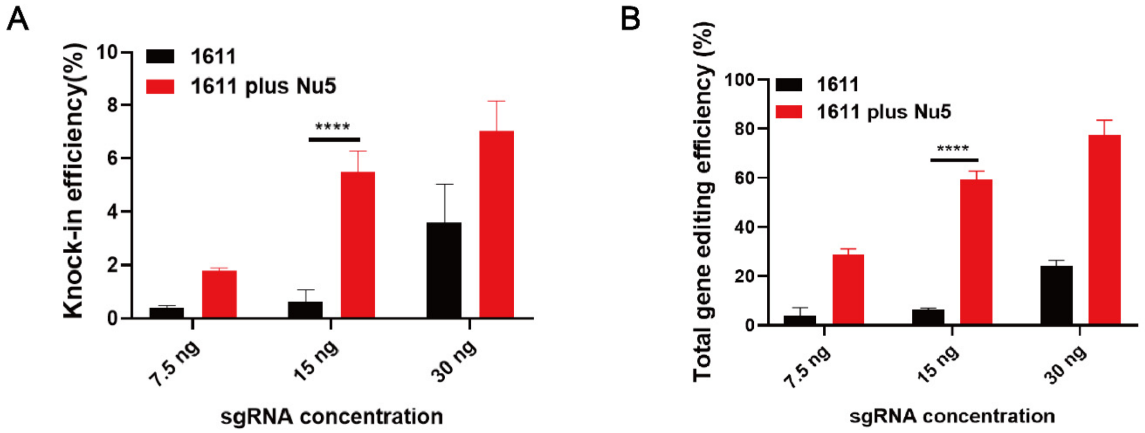

Figure S5. Gene correction and editing of Cas9 mRNA/sgRNA/ssDNA polyplexes with Nu7441 in HeLa-eGFPd2 cells. Cas9 mRNA/sgRNA/ssDNA polyplexes, based on *1611*, were formed at N/P ratio 18 and a dose of 7.5 ng, 15 ng, and 30 ng of total nucleic acid was transfected for 24 h. Data are presented as mean ± SD ($n = 3$). Statistical significance is indicated as **** $p < 0.0001$.

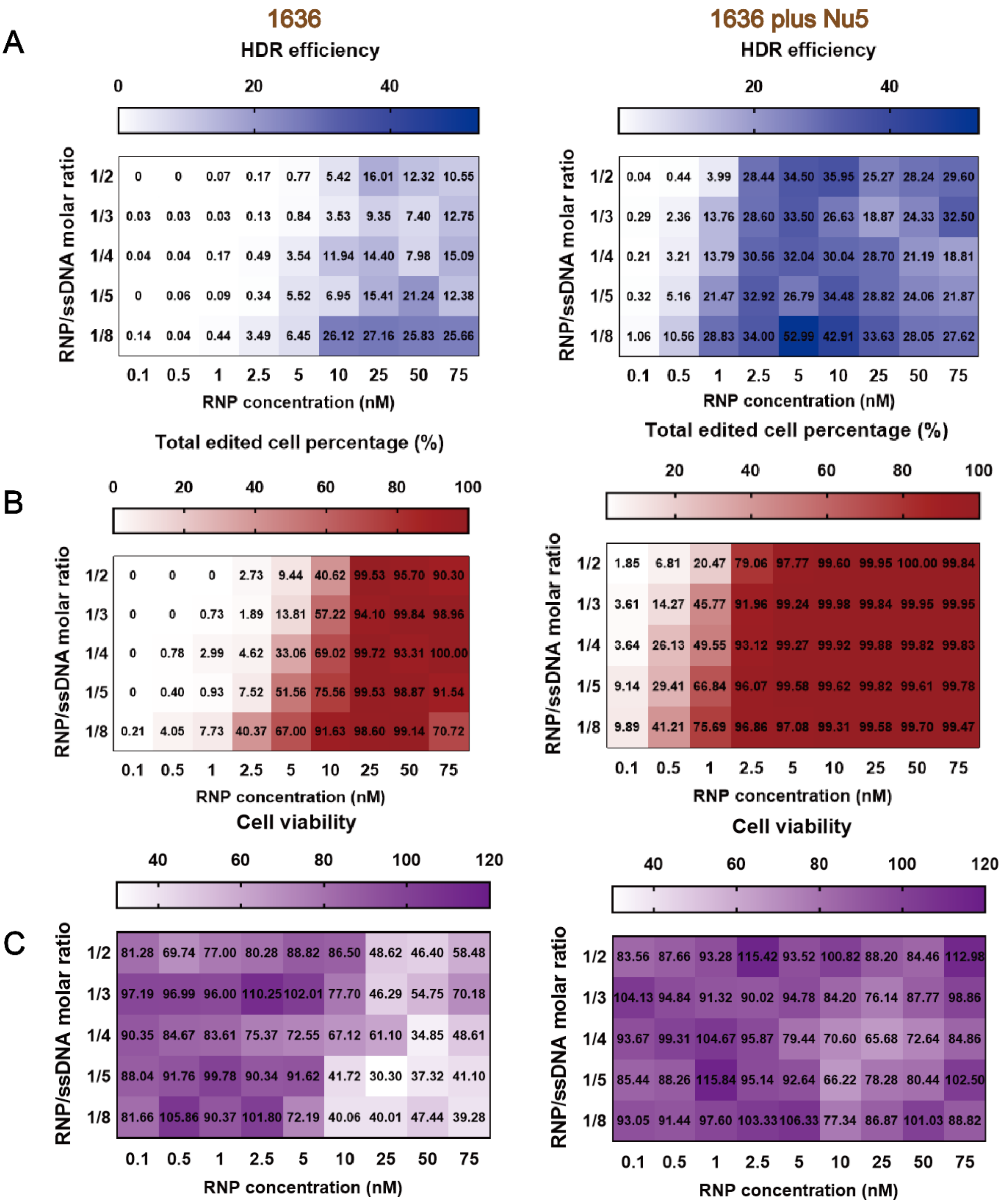

Figure S6. Enhanced HDR-mediated genome editing of HeLa-eGFPd2 applying the optimized HDR T-shape XP (*1636*)-based Cas9 RNP/ssDNA complexes with Nu7441. (**A**) HDR efficiency, (**B**) total gene editing performance, and (**C**) cellular viability were evaluated in HeLa-eGFPd2 cells following treatment with *1636*-based Cas9 RNP/ssDNA complexes at varying sgRNA/ssDNA ratios (1:2, 1:3, 1:4, 1:5, 1:8) and RNP concentrations ranging from 0.1 nM to 75 nM. Figures were labeled with specific values. Cytotoxicity was assessed using the MTT assay. All formulations maintained an N/P ratio of 12. Data are presented as mean (n = 3).

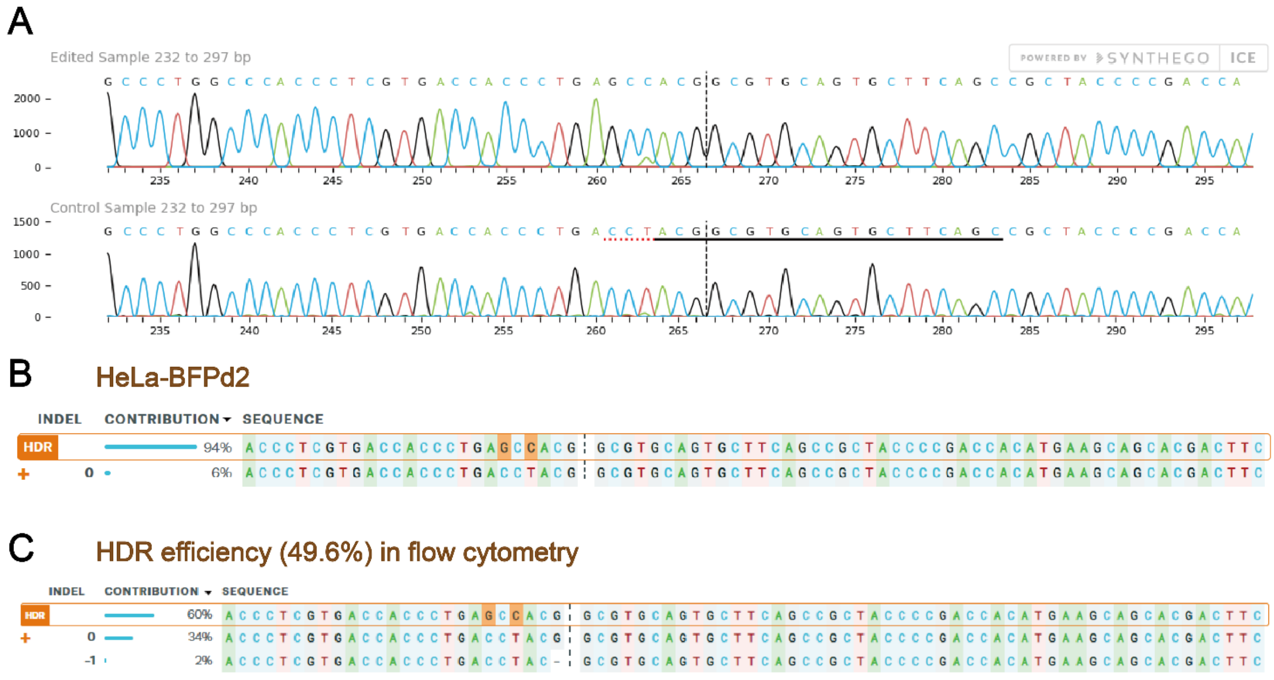

Figure S7. Gene sequence analysis of edited HeLa cells. (**A**) Gene sequence comparison between the edited sample and the untreated control sample. (**B**) Gene sequence analysis of HeLa-BFPd2 cells after single-clone selection. (**C**) Gene sequence analysis of a sample with 49.6% HDR efficiency, as determined by flow cytometry, in the HeLa-eGFPd2 cell model.

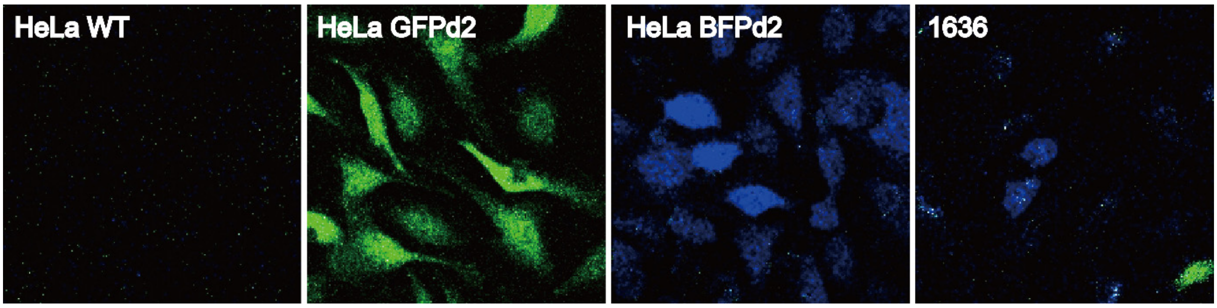

Figure S8. CLSM images of various HeLa cell samples. The CLSM images depicted from standard HeLa cells ('wild-type = WT), HeLa-eGFPd2, and HeLa-BFPd2 cells obtained through single-clone selection, as well as HeLa-eGFPd2 cells following *1636 (1445-Gtt)* treatment.

Supplementary Methods

Cell culture and cell construction

HeLa (ACC57, DSMZ, Germany), HeLa eGFPd2, C2C12 (ACC565, DSMZ, Germany), HeLa mCherry-DMD$_{Ex23}$ (which stably expresses an artificial mCherry construct containing a dystrophin exon 23 sequence interruption derived from the murine Duchenne muscular dystrophy (mdx) model) [1], and HeLa gal8-mRuby3 cells (stably expressing a galectin8-mRuby3 fusion protein) [2,3] were maintained in DMEM supplemented with 10% FBS, 100 U/mL penicillin, and 100 µg/mL streptomycin. Meanwhile, 16HBE14o- (SCC150, Sigma-Aldrich, USA) cells were cultured in MEM with the same supplementation.

To construct the C2C12 eGFPd2, and 16HBE14o-eGFPd2 cell lines, cells were transfected using lipo-XP *1218* polyplex (Table **S3**) [4] or Lipo 3K gene delivery for 48 h. After additional three days of incubation, flow cytometry was performed to sort GFP-positive cells. Subsequently, following an additional seven days of incubation, a second round of sorting was conducted. If the percentage of GFP-positive cells exceeded 20%, cells were seeded at a density of one cell per well in 96-well plates. Finally, single-cell sorting was performed to isolate GFP-positive clones for subsequent experiments. A similar single-cell sorting approach was employed to construct the purified BFP cell line. In these experiments, the reporter cell lines were generated using a PiggyBac transposon system by co-transfecting a transposase-expressing plasmid and a GFP reporter plasmid. To minimize the chance of multiple integrations, a low donor-to-transposase ratio (1:1, W/W) was used. After transfection, monoclonal cell lines were established by single-cell sorting. Flow cytometry analysis showed a single, uniform GFP-positive population without multiple peaks, which is consistent with single-copy integration.

Preparation and transfection evaluation of mRNA/pDNA polyplexes by flow cytometry

The 1611-based mRNA/pDNA polyplex was prepared by mixing mCherry mRNA and pEGFP-N1 at a fixed weight ratio of 1:1, followed by dilution in HBG. The lipo-XP 1611 applied at an N/P ratio of 18 was separately diluted in purified water, then rapidly mixed with the nucleic acid solution and incubated for 40 min at room temperature. The final nucleic acid concentration in the polyplex solution was 12.5 µg/µL. HeLa cells were seeded in 24-well plates at 2.5×10^4 cells per well and cultured for 24 h before transfection. After incubation, the medium was replaced with fresh, pre-warmed medium and polyplex solutions were added to achieve final concentrations of 62.5 ng, 125 ng, or 250 ng of nucleic acid and 50 nM Nu7441 in the cell culture. HBG buffer was used as a negative control.

After 24 h of treatment, cells were collected, resuspended in FACS buffer, and analyzed using a CytoFLEX S Flow Cytometer. DAPI staining was used to distinguish viable from dead cells, with excitation at 405 nm and emission detected at 450 nm. Only isolated viable cells were analyzed. The median fluorescence intensity (MFI) of mCherry- and eGFP-positive cells indicated mRNA and pDNA transfection efficiency, respectively. Fluorescence expression was measured by exciting mCherry at 561 nm and eGFP at 488 nm, with emission detected at 610 nm and 530 nm, respectively.

References

1. Lessl, A.-L.; Pöhmerer, J.; Lin, Y.; Wilk, U.; Höhn, M.; Hörterer, E.; Wagner, E.; Lächelt, U. mCherry on Top: A Positive Read-Out Cellular Platform for Screening DMD Exon Skipping Xenopeptide–PMO Conjugates. *Bioconjugate Chemistry* 2023, *34*, 2263-2274.
2. Rui, Y.; Wilson, D.R.; Tzeng, S.Y.; Yamagata, H.M.; Sudhakar, D.; Conge, M.; Berlinicke, C.A.; Zack, D.J.; Tuesca, A.; Green, J.J. High-throughput and high-content bioassay enables tuning of polyester nanoparticles for cellular uptake, endosomal escape, and systemic in vivo delivery of mRNA. *Science advances* 2022, *8*, eabk2855.
3. Lin, Y.; Wilk, U.; Pöhmerer, J.; Hörterer, E.; Höhn, M.; Luo, X.; Mai, H.; Wagner, E.; Lächelt, U. Folate receptor-mediated delivery of Cas9 RNP for enhanced immune checkpoint disruption in cancer cells. *Small* 2023, *19*, 2205318.
4. Berger, S.; Krhač Levačić, A.; Hörterer, E.; Wilk, U.; Benli-Hoppe, T.; Wang, Y.; Öztürk, O.; Luo, J.; Wagner, E. Optimizing pDNA lipo-polyplexes: a balancing act between stability and cargo release. *Biomacromolecules* **2021**, *22*, 1282-1296.
